# Osimertinib versus afatinib in patients with T790M-positive, non-small-cell lung cancer and multiple central nervous system metastases after failure of initial EGFR-TKI treatment

**DOI:** 10.1186/s12890-021-01539-x

**Published:** 2021-05-19

**Authors:** Yang Yang, Qilong Liu, Lei Cao, Wei Sun, Xiaowei Gu, Bin Liu, Na Xiao, Fei Teng, Xiaoli Li, Meiji Chen, Weiguang Yu, Huanyi Lin, Guixing Xu

**Affiliations:** 1 Hebei Key Laboratory of Cancer Radiotherapy and Chemotherapy, Department of Medical Oncology, Affiliated Hospital of Hebei University, No. 212, Yuhua Dong Road, Lianchi District, Baoding, 071000 Hebei Province China; 2 Department of Gastrointestinal Surgery, The First Affiliated Hospital, Sun Yat-Sen University, No. 58, Zhongshan 2nd Road, Yuexiu District, Guangzhou, 510080 China; 3 Department of Anaesthesiology, The Central Hospital of Wuhan, Tongji Medical College, Huazhong University of Science and Technology, Wuhan, 430014 China; 4 Department of Head and Neck Surgery, Affiliated Hospital of Hebei University, No. 212, Yuhua Dong Road, Lianchi District, Baoding, 071000 Hebei Province China; 5 Central Laboratory, Affiliated Hospital of Hebei University, No. 212, Yuhua Dong Road, Lianchi District, Baoding, 071000 Hebei Province China; 6 Department of Radiotherapy, Affiliated Hospital of Hebei University, No. 212, Yuhua Dong Road, Lianchi District, Baoding, 071000 Hebei Province China; 7 Department of Pediatrics, The First Affiliated Hospital, Sun Yat-Sen University, No. 58, Zhongshan 2nd Road, Yuexiu District, Guangzhou, 510080 China; 8 Department of Orthopaedics, The First Affiliated Hospital, Sun Yat-Sen University, No. 58, Zhongshan 2nd Road, Yuexiu District, Guangzhou, 510080 China; 9 Department of Urinary Surgery, The First Affiliated Hospital, Sun Yat-Sen University, No. 58, Zhongshan 2nd Road, Yuexiu District, Guangzhou, 510080 China; 10 Department of Neurosurgery, The First Affiliated Hospital, Sun Yat-Sen University, No. 58, Zhongshan 2nd Road, Yuexiu District, Guangzhou, 510080 China

**Keywords:** Osimertinib, Afatinib, Non-small-cell lung cancer, Central nervous system, Metastases, Survival

## Abstract

**Background:**

The purpose of this study was to compare the efficacy of osimertinib (OSI) versus afatinib (AFA) in patients with T790M-positive, non-small-cell lung cancer (NSCLC) and multiple central nervous system (CNS) metastases after failure of initial epidermal growth factor receptor tyrosine kinase inhibitor (EGFR-TKI) treatment.

**Methods:**

Consecutive patients with T790M-positive NSCLC and multiple CNS metastases after failure of initial EGFR-TKI treatment were retrospectively identified from our medical institution during 2016–2018 and underwent either oral 80 daily OSI or oral 40 daily AFA every 3 weeks for up to 6 cycles, until disease progression, intolerable adverse events (AEs), or death. The co-primary endpoints were overall survival (OS) and progression-free survival (PFS).

**Results:**

The cohort consisted of 124 patients (OSI: n = 60, mean age = 64.24 years [SD: 12.33]; AFA: n = 64, mean age = 64.13 years [SD: 13.72]). After a median follow-up of 24 months (range, 3 to 28), a significant improvement in OS was detected (hazard ratio [HR] 0.59, 95% confidence interval [CI], 0.39–0.91; *p* = 0.0160; median, 13.7 months [95% CI, 11.1–14.8] for OSI vs 9.6 months [95% CI, 8.4–10.2] for AFA). The median duration of PFS was significantly longer with OSI than with AFA (HR 0.62; 95% CI, 0.41–0.91; *p* = 0.014; median, 4.5 months [95% CI, 3.5–5.7] vs 3.9 months [95% CI, 3.1–4.8]). The proportion of grade 3 or higher adverse events (AEs) was lower with OSI (22.4%) than with AFA (39.4%).

**Conclusions:**

In patients with T790M-positive NSCLC and multiple CNS metastases after failure of initial EGFR-TKI treatment, OSI may be associated with significantly improved survival benefit compared with AFA, with a controllable tolerability profile.

## Background

Osimertinib (OSI; TAGRISSO, AstraZeneca), an oral, 3rd-generation, irreversible epidermal growth factor receptor tyrosine kinase inhibitor (EGFR-TKI) that selectively inhibits both EGFR-TKI-sensitizing and EGFR T790M resistance mutations, has been approved by the Food and Drug Administration (FDA) on November 13, 2015 to treat patients with acquired EGFR T790M resistance or progression on or after EGFR-TKI therapy, and it may resolve the impasse [1, 2]. In a phase I/II clinical trial (AURA Study Phase II Extension Component, NCT01802632) [3] involving 198 evaluable patients with EGFR-TKI–pretreated EGFR- and T790M-positive non-small-cell lung cancer (NSCLC), showed that OSI leads to a promising median progression-free survival (PFS)(12.3 months, 95% confidence interval [CI], 9.5 to 13.8), and median durable response (15.2 months, 95% CI, 11.3 to not calculable). These findings were verified by a Phase II study involving OSI (80 mg/d) in 411 individuals with T790M-positive NSCLC, in which the median PFS was 11.0 months [4]. Nevertheless, whether OSI has superior survival benefits and higher activity against T790M-positive NSCLC and multiple central nervous system (CNS) metastases after failure of initial EGFR-TKI treatment compared with afatinib (AFA) remains unknown [5].

We therefore conducted a retrospective review of patients with T790M-positive NSCLC and multiple CNS metastases after failure of initial EGFR-TKI treatment to compare the efficacy of OSI versus AFA therapy. To our knowledge, this is the first analysis that retrospectively compared OSI against AFA for the management for T790M-positive NSCLC and multiple CNS metastases after failure of initial EGFR-TKI treatment in an Asian population.

## Methods

### Study design and patients

Clinical data of individuals with T790M-positive NSCLC and multiple CNS metastases after failure of initial EGFR-TKI treatment from a registry database were retrospectively identified at the Hebei Key Laboratory of Cancer Radiotherapy and Chemotherapy (Baoding, China) between March 2016 and July 2018. Inclusion criteria: patients with a histologically and/or cytologically confirmed NSCLC harbouring a sensitizing EGFR T790M mutation after failure of initial EGFR-TKI treatment; available NSCLC specimens prior to initial EGFR-TKI treatment; multiple CNS metastases confirmed by imaging evidence (i.e., computed tomography [CT], magnetic resonance imaging [MRI]); patients receiving either oral 80 mg/d OSI or oral 40 mg/d AFA until disease progression, intolerable adverse events (AEs), or death after failure of initial EGFR-TKI treatment; adequate organ function6 [6]; Eastern Cooperative Oncology Group (ECOG) status of 0–2. Key exclusion criteria: previous chemotherapy, radiotherapy, chemoradiotherapy, or surgery for NSCLC and/or CNS metastases; symptomatic CNS metastases at the initial administration of OSI or AFA; severe digestive diseases affecting drug absorption (i.e., perforation and/or fistula formation); insufficient imaging data(lack of CT or MRI data); discontinuation or interruption of OSI or AFA by reason other than AEs; patients receiving OSI and AFA at the same time or in tandem; intolerable AEs (i.e., uncontrolled diabetes or hypertension) that have a significant impact on the co-primary endpoints; severe infections (i.e., HIV infection).

### Outcomes and assessments

Information regarding OSI or AFA delivery, tumour EGFR mutation status, and survival were retrieved from medical records. Tumours were assessed every 4 weeks thereafter by CT, X-rays, bone scans, and MRI as indicated. AEs were evaluated in accordance with the US National Cancer Institute's Patient-Reported Outcomes Version of the Common Terminology Criteria for Adverse Events (PRO-CTCAE) [7]. NSCLC stage was determined according to the Lung Cancer Stage Classification System. Tumour mutation status was tested on plasma or tissue specimens at the State Key Laboratory, Sun Yat-Sen University, as reported [4, 8]. Each patient included in the analysis underwent a re-biopsy after first progression. Overall survival (OS) was calculated from the initiation of drug treatment until the date of death. PFS was calculated from the initiation of drug treatment until the date of disease progression according to RECIST 1.1 or death from any cause, whichever occurred first. Follow-up was executed every 4 weeks during the first 6 month and then every 1 month thereafter. The co-primary endpoints were OS and PFS.

### Statistical analysis

Comparisons of categorical variables between groups were evaluated using Chi-Square tests. Continuous variables were compared with Student t-test for normally distributed variables or Mann–Whitney U test for non- normally distributed variables. Time-to-symptom progression in the subgroup with baseline multiple CNS metastases, median PFS, and median OS were estimated using the Kaplan–Meier method. Comparisons for survival probabilities were assessed using the log-rank test. Survival differences were estimated using the Cox proportional hazard regression model in which partial baseline data (i.e., age, smoking status) were adjusted. The hazard ratio (HR) and corresponding 95% CIs were estimated using Cox proportional hazards models. HRs for PFS and OS were also estimated using a multivariate Cox regression model with adjustment for potential confounding factors. A two-sided *p* value of < 0.05 was considered significant. Data were mainly analysed using SPSS 26.0 (IBM Corp., Armonk, NY).

## Results

### Patients

**Fig. 1:**
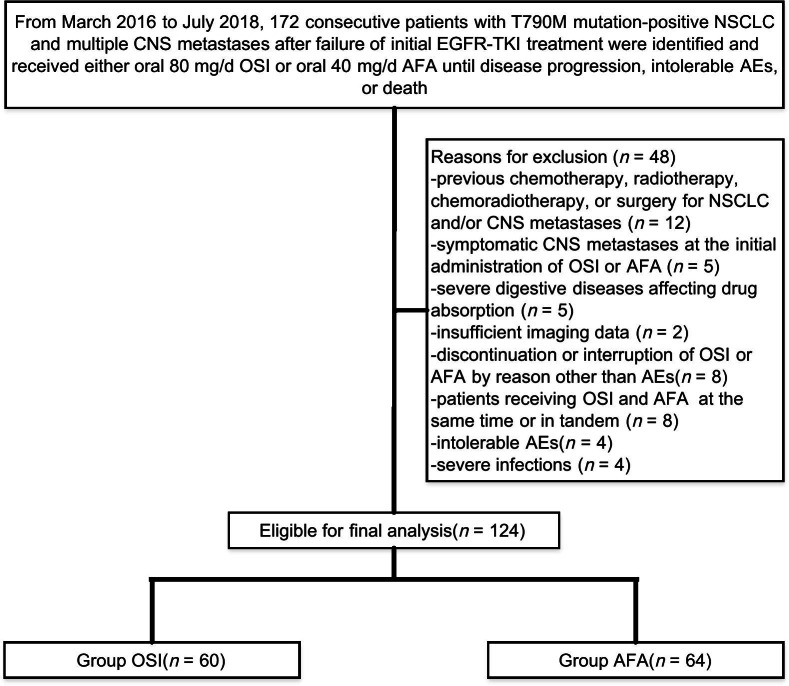
Flow diagram illustrating the methods to retrospectively compare the efficacy of osimertinib (OSI) versus afatinib (AFA) in patients with T790M-positive, non-small-cell lung cancer (NSCLC) and multiple central nervous system (CNS) metastases after failure of initial epidermal growth factor receptor tyrosine kinase inhibitor (EGFR-TKI) treatment

We included a total of 172 patients with T790M-positive NSCLC and multiple CNS metastases after failure of initial EGFR-TKI treatment between March 2016 and July 2018. Forty-eight (27.9%) individuals were excluded on account of the inclusion and exclusion criteria, leaving 124 eligible cases for final analysis, as summarized in Fig. 1 (OSI: n = 60, median age = 64.24 years [range, 51.91 to 76.57]; AFA: n = 64, median age = 64.13 years [range, 50.41 to 77.85]). No significant differences were detected in the demographic data between groups, as presented in Table 1.

**Table 1:** Baseline characteristics

Variable	OSI (n = 60)	AFA (n = 64)	*p *value
Age (years)	64.24 ± 12.33	64.13 ± 13.72	0.72^a^
Sex (male/female), No.%	25/35	28/36	0.82^b^
BMI (kg/m^2^)	23.74 ± 2.31	24.15 ± 2.46	0.26^a^
Smoking status, No.%			0.81^c^
Never a smoker	11	13	
Former smokers	32	30	
Current smokers	17	21	
Time from diagnosis of NSCLC (months), No.%			0.78^c^
< 6	15	17	
6–12	35	33	
> 12	10	14	
Largest size of brain metastasis, No.%			0.31^c^
≤ 10 mm	22	18	
> 10 mm	38	46	
Number of brain metastases, No.%			0.47^c^
≤ 3	32	30	
> 3	28	34	
Previous EGFR-TKI therapy, No.%			0.41^c^
Erlotinib	23	20	
Gefitinib	16	18	
Afatinib	21	26	
Type of EGFR mutation, No.%			0.91^c^
T790M and Exon 19 deletion	35	38	
T790M and Exon 21 L858R	25	26	
Distant metastasis except for CNS, No.%			0.54^c^
Gastrointestinal	22	18	
Pancreas	11	14	
Liver	12	16	
Pancreas	12	14	
Other	3	2	
ECOG performance status, No.%			0.97^c^
0	9	12	
1	27	25	
2	24	27	

*OSI* osimertinib, *AFA* afatinib, *BMI* body mass index, *NSCLC* non-small-cell lung cancer, *EGFR-TKI* epidermal growth factor receptor-tyrosine kinase inhibitor, *CNS* central nervous system, *ECOG* Eastern Cooperative Oncology Group

^a^Analysed using independent-samples t-test

^b^Analysed using Chi-squared test

^c^Analysed using the Mann–Whitney test

### Survival analysis

**Fig. 2:**
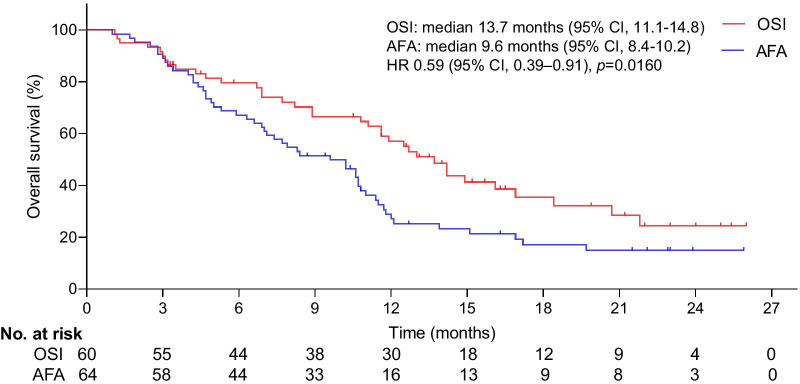
Kaplan–Meier curves for OS. The median OS was 13.7 months (95% CI, 11.1–14.8) for OSI and 9.6 months (95% CI, 8.4–10.2) for AFA. A significant difference was observed in the median OS between groups. *HR was calculated using the Cox proportional hazards model, with age, sex and time span of smoking history as covariates and OSI/AFA therapy as the time-dependent factor. With respect to OS, the results were analysed using the log-rank test (*p* = 0.0160)

**Fig. 3:**
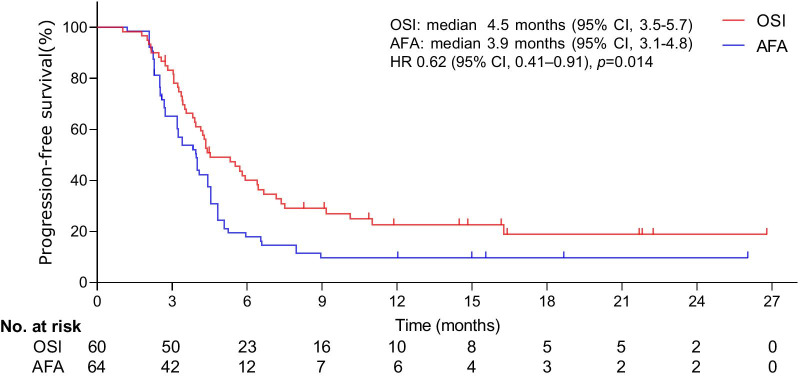
Kaplan–Meier curves for PFS. The median PFS was 4.5 months (95% CI, 3.5–5.7) for OSI and 3.9 months (95% CI, 3.1–4.8) for AFA. A significant difference was detected in the median PFS between groups. *HR was calculated using the Cox proportional hazards model, with age, sex and time span of smoking history as covariates and OSI/AFA therapy as the time-dependent factor. With respect to PFS, the results were analysed using the log-rank test (*p* = 0.014)

The median duration of follow-up was 24 months (range, 3 to 28). At the final follow-up, the median duration of OS was 13.7 months (95% CI, 11.1–14.8) in the OSI group and 9.6 months (95% CI, 8.4–10.2) in the AFA group. The main cause of death in patients tends to coma induced by CNS metastases. The median duration of OS was significantly longer with OSI than with AFA (HR 0.59; 95% CI, 0.39–0.91; *p* = 0.0160; Fig. 2). The median duration of PFS was 4.5 months (95% CI, 3.5–5.7) in the OSI group and 3.9 months (95% CI, 3.1–4.8) in the AFA group. The median duration of PFS was significantly longer among patients undergoing OSI than among those treated with AFA (HR 0.62; 95% CI, 0.41–0.91; *p* = 0.014; Fig. 3).

### Adverse events

Safety was assessed for each patient treated with OSI or AFA. AEs occurred in 48 of 60 patients (80.0%) in the OSI group and in 59 of 64 (92.2%) in the AFA group. Drug-related AEs were summarized in Table 2. The proportion of grade 3 or higher AEs was lower with OSI (22.4%) than with AFA (39.4%). Most AEs were mild-to-moderate in severity and reversible. In our study, there was not significant difference in the hematological toxicity between groups, although it is one of the major side effects of EGFR-TKI.

**Table 2:** Drug-related adverse events

Variable	OSI (n = 85 AEs involving 48 patients)			AFA (n = 109 AEs involving 59 patients)		*p* value^*a*^	
		All grades (%)	≥ Grade 3 (%)	All grades (%)	≥ Grade 3 (%)	All grades (%)	≥ Grade 3 (%)
Diarrhoea		24 (28.2)	8 (9.4)	47 (43.1)	25 (22.9)	0.03*	0.13
Rash		22 (25.9)	5 (5.9)	18 (16.5)	7 (6.4)	0.11	0.88
Dry skin		18 (21.2)	6 (7.1)	12 (11.0)	5 (4.6)	0.52	0.46
Paronychia		11 (12.9)	0 (0.0)	6 (5.5)	0 (0.0)	0.07	NA
Alopecia		3 (3.5)	0 (0.0)	15 (13.8)	6 (5.5)	0.02*	0.03*
Asthenia		2 (2.4)	0 (0.0)	6 (5.5)	0 (0.0)	0.27	NA
Decreased appetite		5 (5.8)	0 (0.0)	5 (4.6)	0 (0.0)	0.69	NA

The OSI-related AEs that were most commonly reported included diarrhoea (24 patients [28.2%]; 8 [9.4%] were deemed as ≥ grade 3), rash (in 22 [25.9%]; 5 [5.9%] were ≥ grade 3), dry skin (in 18 [21.2%]; 6 [7.1%] were deemed as ≥ grade 3), and paronychia (in 11 [12.9%]; no one was deemed as ≥ grade 3). Most rashes were regarded as grade 1 or 2 (17 [20.0%] for OSI vs. 11 [10.1%] for AFA; *p* = 0.05). The most common AFA-related AEs were diarrhoea (47 patients [43.1%]; 25 [22.9%] were deemed as ≥ grade 3), alopecia (in 15 [13.8%]; 6 [5.5%] were ≥ grade 3), asthenia (in 6 [5.5%]; no one was deemed as ≥ grade 3), decreased appetite (in 5 [4.6%]; no one was deemed as ≥ grade 3) and rash (in 18 [16.5%]; 7 [6.4%] were deemed as ≥ grade 3).

## Discussion

Findings from this retrospective analysis comparing the efficacy of OSI versus AFA as second-line treatment in patients with T790M-positive NSCLC and multiple CNS metastases after failure of initial EGFR-TKI treatment, showed that despite the short follow-up, OSI might be associated with significantly improved survival benefit compared with AFA for patients with T790M-positive NSCLC and multiple CNS metastases, with a controllable tolerability profile. The underlying background and reason of the shorter survival could be the fact that patients with T790M-positive NSCLC and multiple CNS metastases tend to have a worse prognosis than those who have no such metastases.

Previous reports [3, 8–11] of OSI or AFA in T790M-positive NSCLC have encountered unexpected obstacles, including non-uniform definitions of variables, undefined CNS metastases, and discontinuous medication, which may lead to survival variability. Although the limited sample size makes it problematic to present convincing conclusions in the current analysis, the survival advantage of OSI is more significant compared with that of AFA, but further validation for our findings was required. The key limiting factor for such cohort is to grasp the timing of multiple CNS metastases. For PFS, a well-defined finding favouring the OSI regimen was described [3, 11], although PFS was not the setting of the primary endpoint. The reason why PFS but not OS was affected could be associated with small cohort of research subjects with NSCLC progression who failed to undertake EGFR-TKI treatment [12].

The evidence-based trials [9, 13] regarding the optimal regimen in the management of cases with T790M-positive NSCLC and multiple CNS metastases remain controversial. Within this context, evidence has indicated that neither the first-generation nor second-generation EGFR-TKIs distinctly improve the OS among those cases with T790M-positive NSCLC [9, 14]. Nonetheless, whether there were brain metastases in the included cohorts and whether multiple CNS metastases in the studied cohorts and whether CNS metastases offset some of the EGFR-TKI efficacy remain ambiguous [14, 15], which could make the facts further obscured. There is a significant difference in the composition ratio of patients with multiple CNS metastases in each group, possibly resulting from variability in the response to the EGFR-TKI in diverse reports [5, 16]. In the present study, the survival benefits of OSI over AFA were consistent with prospective randomized trials [11, 17–19] in which the composition ratio of multiple CNS metastases was assessed in subgroup analyses. Consequently, the between-group differences in survival benefits might be attributed to drug mechanistic differences [18, 20]. It is a well-established fact that OSI has a higher penetrance to the brain than 1st or 2nd generation TKIs, which tends to be the most important explanation for the better outcome for patients receiving OSI, in addition to the fact that it has better results in general for T790M-positive patients [21–23].

However, consensus is lacking as to what is the optimal treatment regimen for such cohorts [4, 20, 24]. At present, progressing NSCLC in patients who have undertaken first- or second-generation EGFR-TKIs is best treated with OSI or its combination with chemotherapy if feasible [3, 4, 25]. Although OSI is not part of the current standard of care in China, it was approved by the FDA for the management of the T790M-positive NSCLC patients [18, 26]. Previous studies [25, 27] have confirmed that the irreversible inhibitor OSI offers more survival benefits compared with other reversible EGFR-TKIs. Of note, those studies in which some subjects failed to be included based on the T790M mutation appear to have unclear data or to have restricted subjects according to the researcher's preference. Additionally, OSI has been less frequently used because the empirical use of drugs is common in clinical practice despite the lack of reliable supportive evidence in the first few years [3, 18, 28].

Although our analysis contributes to gaining a better understanding of the survival benefits of the continued OSI treatment in the setting of T790M-positive NSCLC and multiple CNS metastases after failure of initial EGFR-TKI treatment, there are certain limitations to discuss. First, the level of evidence in the current study is limited due to the weaknesses inherent in a retrospective analysis including treatments, follow-up, and missing data. A number of cases were excluded at the final follow-up owing to imperfect follow-up data, which may introduce bias. The capacity to draw reliable conclusions may be reduced due to the biases that may have contributed to differences in outcomes. Second, the current outcomes were limited by the follow-up protocol (i.e. frequency, length). Third, possible heterogeneity seems hard to avoid, even though considerable variables have been adjusted.

## Conclusions

Our study demonstrated that a noteworthy survival superiority of OSI over AFA was observed in patients with T790M-positive NSCLC and multiple CNS metastases after failure of initial EGFR-TKI treatment. AFA is a broad HER-family inhibitor, whereas OSI is a highly specific EGFR-inhibitor directed towards the sensitizing mutations and a potent inhibitor of the T790M mutation. The latter is probably most important in this resistance setting, along with the better CNS-penetrance. We believe that OSI could be a more effective therapeutic option than AFA in the current setting. Although our analysis was powered to assess end-points, given high mortality associated with T790M mutation-positive NSCLC, if OSI or AFA were to be re-evaluated in the comparable setting, extended follow-up time is necessitated to clarify whether our findings persist over an extended follow-up.

## Data Availability

The datasets used and/or analysed during the current study are available from the corresponding author upon reasonable request.

## Abbreviations

OSI: Osimertinib
AFA: Afatinib
NSCLC: Non-small-cell lung cancer
EGFR-TKI: Epidermal growth factor receptor tyrosine kinase inhibitor
AEs: Adverse events
OS: Overall survival
PFS: Progression-free survival
SD: Standard deviation
CI: Confidence interval
IQR: Interquartile range
HR: Hazard ratio
T790M: P.Thr790Met
CT: Computed tomography
MRI: Magnetic resonance imaging
ECOG: Eastern Cooperative Oncology Group
VCI: Vascular cognitive impairment
RECIST: Response Evaluation Criteria in Solid Tumours
